# Effect of Androgen Deprivation Therapy on Other-Cause of Mortality in Elderly Patients with Clinically Localized Prostate Cancer Treated with Modern Radiotherapy: Is There a Negative Impact?

**DOI:** 10.3390/jcm8030338

**Published:** 2019-03-11

**Authors:** Hideya Yamazaki, Koji Masui, Gen Suzuki, Satoaki Nakamura, Norihiro Aibe, Daisuke Shimizu, Kei Yamada, Koji Okihara, Takumi Shiraishi, Tadayuki Kotsuma, Ken Yoshida, Eiichi Tanaka, Keisuke Otani, Yasuo Yoshioka, Kazuhiko Ogawa, Tatsuyuki Nishikawa, Haruumi Okabe

**Affiliations:** 1 Department of Radiology, Graduate School of Medical Science, Kyoto Prefectural University of Medicine, 465 Kajiicho Kawaramachi Hirokoji, Kamigyo-ku, Kyoto 602-8566, Japan; mc0515kj@koto.kpu-m.ac.jp (K.M.); gensuzu@koto.kpu-m.ac.jp (G.S.); satoaki@nakamura.pro (S.N.); a-ib-n24@koto.kpu-m.ac.jp (N.A.); dshimizu@koto.kpu-m.ac.jp (D.S.); kyamada@koto.kpu-m.ac.jp (K.Y.); 2 Department of Urology, Graduate School of Medical Science, Kyoto Prefectural University of Medicine, 465 Kajiicho Kawaramachi Hirokoji, Kamigyo-ku, Kyoto 602-8566, Japan; kokihara@koto.kpu-m.ac.jp (K.O.); takumi14@koto.kpu-m.ac.jp (T.S.); 3 Department of Radiation Oncology, National Hospital Organization Osaka National Hospital, 2-1-14, Hoenzaka, Chuo-ku, Osaka 540-0006, Japan; tkotsuma-osaka@umin.net (T.K.); rad113@osaka-med.ac.jp (K.Y.); tanaka@onh.go.jp (E.T.); 4 Department of Radiation Oncology, Osaka University Graduate School of Medicine, Suita, Osaka 565-0871, Japan; ohtanik@radonc.med.osaka-u.ac.jp (K.O.); yasuo.yoshioka@jfcr.or.jp (Y.Y.); kogawa@radonc.med.osaka-u.ac.jp (K.O.); 5 Department of Radiology, Ujitakeda Hospital, Uji-City, Kyoto 611-0021, Japan; tnishikawa201809@yahoo.co.jp (T.N.); h-okabe@takedahp.or.jp (H.O.)

**Keywords:** prostate cancer, androgen deprivation therapy, brachytherapy, IG-IMRT, elderly, OCM

## Abstract

The influence of androgen deprivation therapy (ADT) on other-cause of mortality (OCM) was investigated in patients with localized prostate cancer treated with modern high-dose radiotherapy. A retrospective review was conducted on 1125 patients with localized prostate cancer treated with high-dose radiotherapy, including image-guided, intensity-modulated radiotherapy or brachytherapy with a median follow-up of 80.7 months. Overall survival rate was no different between ADT (+) and ADT (−) group in high-, intermediate-, and low-risk groups. OCM was found in 71 patients, consisting of 4% (10/258) in the ADT (−) group and 7% (61/858) in the ADT (+) group (*p* = 0.0422). The 10-year OCM-free survival rate (OCMFS), if divided by the duration of ADT (ADT naïve (ADT (−)), ADT <2-year, and ADT ≥2-year groups), showed statistical significance, and was 90.7%, 88.2%, and 78.6% (*p* = 0.0039) for the ADT (−), ADT <2-year, and ADT ≥2-year groups, respectively. In patients aged ≥75 years, 10-year OCMFS for ADT (−), ADT <2-, and ADT ≥2-year groups was 93.5% (at 115.6 months), 85.6%, and 60.7% (*p* = 0.0189), respectively, whereas it was 90.7%, 89.9%, and 89.0% (*p* = 0.4716), respectively, in their younger counterparts. In localized prostate cancer patients, treatment with longer ADT for ≥2 years potentially increases the risk of OCM, especially in patients aged ≥75 years.

## 1. Introduction

Prostate cancer is one of the most frequently diagnosed cancers in men in developed countries [1,2]. Its standard treatment options are radical prostatectomy, external beam radiotherapy (EBRT), and interstitial brachytherapy (BT) [3]. Androgen deprivation therapy (ADT) is another important treatment, and a number of well-designed prospective multicenter trials have confirmed the value of ADT in addition to the standard radiation therapy [4,5]. Although modern radiotherapy routinely delivers higher biological equivalent doses (BEDs) in excess of 74 Gy to the prostate, decisions on the use of additional ADT in high-BED modern radiotherapy have proved inconclusive since a randomized trial to confirm the role of ADT therapy was performed with up to 70 Gy of EBRT [3,6]. Accordingly, guidelines recommended additional ADT in high-risk patients; however [3], limited evidence supports these recommendations of higher BED use in modern radiotherapy (including image-guided, intensity-modulated radiotherapy (IG-IMRT) and BT) [7]. Furthermore, ADT has several untoward effects, such as cardiovascular toxicity and diabetes; therefore, fragile patients with multiple comorbidities underwent DT with meticulous caution [8,9] since it is reported to be a potential influencing factor of other-cause mortality (OCM) caused by factors other than prostate cancer [8,9]. As a patient database comprising >1000 BT and IG-IMRT patients had been established for over a decade [10], the merit of ADT was examined in the modern radiotherapy era. In addition, the influence of ADT on the aged population was also investigated because elderly patients risked having a fragile health status with multiple comorbidities [9,10]. Further, this study aimed to examine the role of ADT in men who had been diagnosed with localized prostate cancer and were treated with modern high BED radiotherapy, with a focus on the age factor.

## 2. Methods

### 2.1. Patients

Data of 1125 patients who were treated with modern, high-BED radiotherapy between 1995 and 2013 were retrospectively examined. The eligibility criteria were patients who had been treated with high-dose-rate brachytherapy (HDR-BT) monotherapy or low-dose-rate brachytherapy (LDR-BT) with or without EBRT or IG-IMRT with a curative intent; a clinical stage T1–T4 and N0, M0 with histology-proven adenocarcinoma; availability and accessibility of data on determining the National Comprehensive Cancer Network (NCCN) risk classification (pretreatment prostate-specific antigen (initial PSA = iPSA) level, Gleason score sum (GS), and/or T classification); and 1-year minimum follow-up for surviving patients or until death. Among the 1128 included patients, three were excluded due to a lack of follow-up after <1 year or because of missing data. Thus, the final study included 1125 patients as subjects.

A total of 867 ADT (+) patients with stage T1–T4, N0, M0 prostate cancer were treated with radiotherapy as compared to 258 ADT (−) patients. Patients’ clinical characteristics are shown in Table 1. Patients were staged according to the NCCN 2015 risk classification as follows: low risk: T1–T2a and GS 2–6 and iPSA level <0 ng/mL; intermediate risk: T2b–T2c or GS 7 or PSA level 10–20 ng/mL; and high risk: T3a–T4 or GS 8–10 or PSA level >20 ng/mL [3]. PSA failure was defined using the Phoenix definition (nadir, +2 ng/mL) or as the start of salvage hormonal therapy. The Common Terminology Criteria for Adverse Events version 4.0 Toxicity was used for toxicity analysis. All patients provided informed written consent. This study was conducted in accordance with the Declaration of Helsinki, and Institutional Review Board permission was obtained from each institution.

### 2.2. Treatment Planning

#### 2.2.1. Brachytherapy (BT)

BT contained low-dose-rate interstitial BT (LDR-BT) with or without external beam radiotherapy (EBRT) and high-dose-rate interstitial BT (HDR-BT) monotherapy.

##### Low-Dose-Rate Interstitial BT (LDR-BT) with or without EBRT

The implant technique has been previously described in detail elsewhere [11,12]. All patients underwent transrectal ultrasound (TRUS) preplanning 3–4 weeks before implantation to determine the number of seeds. Moreover, intraoperative permanent I-125 implantation (The OncoSeed model 6711; General Electric Healthcare, Barrington, IL, USA) was performed using modified peripheral loading method. Our prescription dose for the clinical target volume (prostate) was 145 Gy (ADT (−) only) or 110 Gy (LDR-BT with EBRT). Inter-Plan version 3.4 (Elekta, Stockholm, Sweden) was used as the treatment planning system. A combination therapy was administered for ≤T3 or a GS ≤8, or for a GS of 7 (4 + 3) cases (not for GS of 7 (3 + 4) cases). Our prescription dose for the clinical target volume (CTV) (prostate) was 145 Gy (LDR-BT only) or 110 Gy (LDR-BT with EBRT) (2005–2013). The ADT was used in patients for >6 months before and/or immediately after brachytherapy [11]. In men with prostate volume >40 cc, ADT was selected to reduce the prostate volume.

##### High-Dose-Rate Interstitial Brachytherapy (HDR-BT) Monotherapy

The detailed technique used has been previously described elsewhere [13,14]. Briefly, a simple, radiography-based treatment planning was used from 1995 to 2007, and the prescription dose point was positioned 5 mm away from a source in the central plane. This method was denoted two-dimensional planning and was used as the initial treatment. The dose point was then shifted from two- to three-dimensional (3D) planning, and the remaining patients were treated with this (i.e., computed tomography (CT)-based planning). For 3D planning, the D90 and D95 or more were used to evaluate adequate coverage of the planning target volume. The CT-based planning with or without magnetic resonance imaging (MRI) assistance was performed by computer optimization (Nucletron an Elekta Company, Veenendaal, The Netherlands, PLATO^®^ and Oncentra^®^ brachy, Elekta AB, Stockholm, Sweden) with or without manual modification. The primarily prescribed dose was 45.5 Gy per 7 fractions, 54 Gy per 9 fractions for 5 days, 49 Gy per 7 fractions, and others (36–38 Gy per 4 fractions) [13,14]. The treatment machine used was the microSelectron-HDR^®^ (Nucletron an Elekta Company, Veenendaal, The Netherlands). For ADT administration, nearly all patients first received both neoadjuvant and adjuvant ADT for two or three years or a lifetime [14]. Second, the duration of ADT for intermediate-risk patients was shortened to 6–12 months, and ADT was administered mainly as neoadjuvant therapy. Third, all patients underwent a total of 12 months of ADT. For our most recent protocol, patients with only one intermediate-risk feature were not administered ADT, whereas all the others underwent 6 months as neoadjuvant but no adjuvant ADT [14].

#### 2.2.2. IG-IMRT

We used helical tomotherapy for IG-IMRT; the details have been described elsewhere [15,16]. Briefly, CT with a slice thickness of 2 mm in a supine position and MRI data (T1w and T2w) were employed in precise radiotherapy planning. The CTV was defined as the prostate and proximal seminal vesicles or prostate only in the low-risk group (Damico’s classification: stage, T1c; Gleason score <7; and PSA level <10 ng/mL). We started IG-IMRT using a 2.2 Gy fraction schedule with D95 (95% of pllaning target volume received at least the prescribed dose) of 74.8 Gy in 34 fractions for intermediate- and high-risk patients and 72.6 Gy in 33 fractions for the low-risk patients (June 2007 to 2009). The prescribed dose was modified by reducing to 74 Gy (D95) in 37 fractions for the high- and intermediate-risk groups, and 72 Gy in 36 fractions for the low-risk group (2 Gy/fraction) from June 2009 to September 2013. ADT was administered 3–6 months for neoadjuvant therapy and 12–24 months for adjuvant therapy in general according to their risk factors.

### 2.3. Statistical Analysis

StatView 5.0 statistical software was used for statistical analyses. Percentages were analyzed using a chi-square test, and student’s *t*-test was used for normally distributed data. The Mann–Whitney *U*-test for skewed data was used to compare means or medians. The Kaplan–Meier method was used to analyze survival, and comparisons were made using the log-rank test. *p* < 0.05 was considered as statistically significant.

## 3. Results

### 3.1. Patients’ Characteristics

The median follow-up for the entire cohort was 80.7 (ranging from 5 to 241) months, with a minimum of 1 year in surviving patients or until death.

A comparison of the background characteristics between ADT (+) and ADT (−) group is shown in Table 1. ADT (+) patients included those with advanced disease (higher T category, higher initial PSA level, higher Gleason score sum, and higher risk group in NCCN risk classification), and also underwent a higher proportion of IG-IMRT than BT. Elderly patients underwent more ADT with borderline significance.

### 3.2. Overall Survival (OS) and OCM According to ADT Usage

Actuarial 10-year OS rate was 90.7% (95% confidential interval (CI): 82.9–98.6%) in ADT (−) and 86.7% (83.2–90.2%) in ADT (+) group (*p* = 0.1202), respectively; it was 78.3% (74.6% for ADT (−) and 78.6% for ADT (+), *p* = 0.6773) for the high-risk group, 91.9% (97% for ADT (−) and 91% ADT (+), *p* = 0.22008) for the intermediate-risk group, and 92.4% (89.2% ADT (−) and 93.5% ADT (+), *p* = 0.6031) for the low-risk group. Statistically significant differences were observed among the three risk groups (*p* < 0.0001) in terms of OS. Furthermore, we compared the OS among three ADT groups divided by the duration of ADT (ADT naïve, ADT <2 years and ADT ≥2 years). A statistically significant difference was also observed among the three groups, which was 90.1%, 88.4%, and 78.5% (*p* = 0.0045) for theADT (−), ADT <2-year, and ADT ≥2-year groups, respectively. Table 2 shows associations between mortality and ADT subgroup.

OCM was found in 71 patients, consisting of 4% (10/258) in ADT (−) group and 7% (61/858) in ADT (+) group (*p* = 0.0422) (Table 3). Table 3 shows the association between OCM and influential factors. Age, advanced disease (higher T category, higher initial PSA level, higher risk group in NCCN classification), ADT use, and modalities used were the significant influencing factors for OCM.

Actuarial 10-year OCM-free survival rate (10 year-OCMFS) was 90.7% (95% CI: 82.9–98.6%) in the ADT (−) and 87.9% (84.5–91.3%) in the ADT (+) group (*p* = 0.1688, Figure 1), while it was 81.1% (74.6% for ADT (−) and 81.7% for ADT (+), *p* = 0.9773) for the high-risk group, 91.9% (97% for ADT (−) and 91% ADT (+), *p* = 0.2208) for the intermediate-risk group, and 92.4% (89.2% for ADT (−) and 93.5% ADT (+), *p* = 0.6031) for the low-risk group. A statistically significant difference was observed among the three groups (*p* = 0.0013).

**Figure 1:**
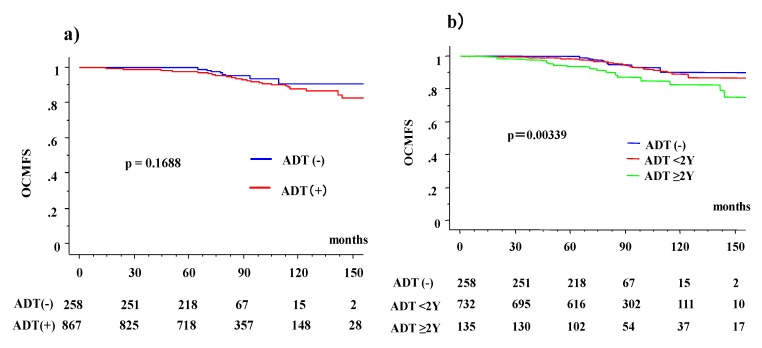
Other cause of mortality (OCM) with or without androgen deprivation therapy (ADT). (**a**) OCM according to ADT (+) and (−) in total population. (**b**) OCM according to three ADT groups divided by duration (ADT naïve, ADT <2 years and ADT ≥2 years). OCMFS = other cause of mortality (OCM)-free survival rate.

If divided by the duration of ADT (ADT (−), ADT <2-year, and ADT ≥2-year groups), a statistically significant difference was observed (Table 4); from the 2% OCM ratio found in the ADT (−) group, it increased to 6% (ADT <2-year) and 13% in the ADT ≥2-year groups (*p* = 0.0007). The 10-year OCMFS was 90.7%, 88.2%, and 78.6% (*p* = 0.0039, Figure 1) for the ADT (−), ADT <2-year, and ADT ≥2-year groups, respectively. The values were 74.6%, 81.5%, and 81.2% (*p* = 0.5689) for ADT (−), ADT <2-year, and ADT ≥2-year groups, respectively, in the high-risk group; 97.0%, 90.8%, and 92.3% (*p* = 0.4031) in the intermediate-risk group; and 89.2%, 93.5%, and not available (*p* = 0.6031) in the low-risk group.

We experienced 72 biochemical failures after radiotherapy. Among them, only one patient (1/72 = 1%) showed OCM, whereas 71 who did not experience biochemical failure showed OCM (71/1115 = 7%, *p* = 0.1272). As ten prostate-cancer-related deaths were reported in this cohort (Table 1, 9 high-risk and one intermediate-risk patient all underwent HDR-BT monotherapy with ADT), the 10-year prostate cancer cause-specific survival rates were 98.8% (98.5% in ADT (+) and 100% in ADT (−), *p* = 0.5080).

### 3.3. OS and OCM between ADT (−) and ADT (+) Patients According to Age

A comparison of background patient characteristics between elderly patients and their younger counterparts is displayed in Table 5. Elderly patients developed advanced diseases and required more ADT treatment by external beam radiotherapy. It is natural that patients aged ≥75 years showed poorer OS than their younger counterparts (10-year OS rate = 88.9% vs. 82.8%, *p*<0.0001). This is further enhanced when comparing the OS in three ADT groups divided by the duration of ADT (ADT naïve, ADT <2 years and ADT ≥2 years). The 10-year OS rate (at 115.6 months) were 93.5%, 85.6%, and 58.2% (*p* = 0.005) for ADT (−), ADT <2 years, and ADT ≥2 years groups, respectively, in the elderly population. In contrast, no statistically significant difference was found in the younger counterpart, showing 90.7%, 89.2%, and 84.5%, respectively (*p* = 0.1744).

OCM gradually increases with age (Table 2). Regarding the patients aged ≥75 years, 10.6% (26/244) experienced OCM, whereas 5.1% (45/881) of patients experienced OCM in the younger group (*p* = 0.0016, Table 4); this was also true regarding the assessment of details. Only a 2% OCM ratio was found in those aged up to age 59 years, increasing to 4% (aged 60–69 years), 7% (aged 70–74 years), and 11% in those aged ≥75 years (*p* = 0.0017). Actuarial 10-year OCMFS were 89.9% (95% CI: 86.6–93.3%) and 83.2% (95% CI: 75.9–90.6%) in the elderly population and in their younger counterpart (*p* < 0.0001), respectively.

In assessing the ADT influence, especially in the patients aged ≥75 years, 12.1% (24/198) of patients experienced OCM in the ADT (+) group, whereas 4.3% (2/46) experienced OCM in the ADT (−) group (*p* = 0.1237, Table 3). In the younger population, 3.3% (7/212) of patients experienced OCM in the ADT (−) group and 5.68% (38/669) in the ADT (+) group (*p* = 0.1705). Comparing the three groups (ADT (−), ADT <2-year, and ADT ≥2-year) (Table 3), a statistically significant correlation was found in the elderly patients aged ≥75 years (4%, 10%, and 24% OCM for the ADR (−), ADT <2-year, and ADT ≥2-year groups, respectively, *p* = 0.0185), whereas borderline significance was observed in their younger counterparts (3%, 5%, and 10% of OCM for the ADR(−), ADT <2-year, and ADT ≥2-year groups, respectively, *p* = 0.0439).

**Figure 2:**
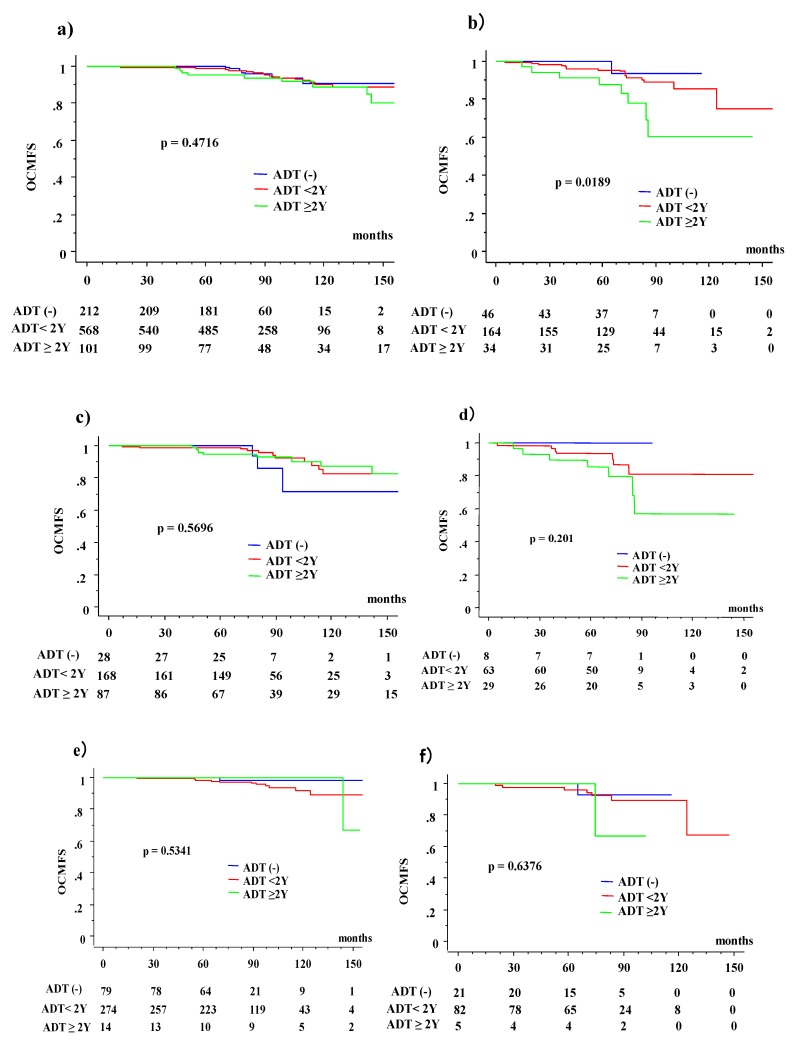

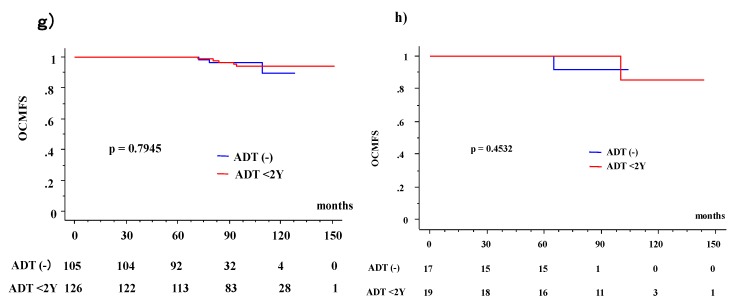
Other-cause of mortality (OCM) with or without androgen deprivation therapy (ADT) according to age. (**a**) OCMFS according to three ADT groups divided by duration (ADT naïve, ADT <2 years (2Y), and ADT ≥2 years (2Y)) in younger population. (**b**) OCMFS according to three ADT groups divided by duration in elderly population. (**c**) OCMFS according to three ADT groups divided by duration in high-risk group of younger population. (**d**) OCMFS according to three ADT groups divided by duration in the high-risk group of the elderly population. (**e**) OCMFS according to three ADT groups divided by the duration in the intermediate-risk group of the younger population. (**f**) OCMFS according to three ADT groups divided by the duration in the intermediate-risk group of the elderly population. (**g**) OCMFS according to three ADT groups divided by the duration in the low-risk group of the younger population. (**h**) OCMFS according to three ADT groups divided by the duration in the low-risk group of the elderly population. OCMFS = other-cause of mortality (OCM)-free survival rate.

The 10-year OCMFS were 89.8% and 90.7% for the ADT (−) and ADT (+) groups (*p* = 0.5688), respectively, in the younger population, whereas they were 93.5% at 115.6 months and 81.5% for the ADT (−) and ADT (+) groups (*p* = 0.2451), respectively, in the elderly population (Figure 2). These differences were enhanced by dividing the analysis into three ADT groups (ADT (−), ADT <2-year, and ADT ≥2-year groups), which were 93.5% (at 115.6 months), 85.6%, and 60.7% (*p* = 0.0189) in the elderly group, and 90.7%, 89.9%, and 89.0% (*p* = 0.4716) for ADT (−), ADT <2-year, and ADT ≥2-year groups in their younger counterpart, respectively.

The 10-year OCMFS among the three ADT groups in the NCCN risk group classifications (Figure 2) are as follows: 71.8%, 82.3%, and 87.2% (*p* = 0.5696) for ADT (−), ADT <2-year, and ADT ≥2-year groups in the younger population, respectively; 100%, 80.9%, and 56.8% (*p* = 0.201) in the elderly population, respectively; 89%, 91.6%, and 100% (*p* = 0.5341) in the younger group, respectively; 92.9% at 115.6 months, 89.8% and 66.7% at 102 months (*p* = 0.6376) in the intermediate-risk group; and 89.8%, 97.3% (*p* = 0.7945), and not available in the younger group, 91.7% at 10.4 months, and 85.7% and not available (*p* = 0.4532) in the low-risk group.

### 3.4. Causes of OCM

Table 6 depicts the cause of OCM. Other cancer forms are a major cause of OCM. In the ADT (−) group, no cardiovascular death was recorded, whereas 0.4% and 1.5% mortality (due to cardiovascular death) was recorded in the ADT <2-year and ADT ≥2-year groups, respectively, although no statistically significant difference was observed. Unknown cases included three cases of sudden death that did not exclude cardiovascular death.

## 4. Discussion

We presented here that ADT did not always improve outcomes after high-BED radiotherapy for localized prostate cancer patients. In fact, long-term ADT ≥2-year may have a negative impact on OCM, especially in elderly patients aged ≥75.

ADT has played an important role in the management of prostate cancer. In the early 1940s, Huggins and Hodges [17,18] established how castration arrested the growth of prostate cancer cells and suppressed serum prostate phosphatases in metastatic prostate cancer cells. Following several randomized controlled trials, simultaneous radiotherapy with ADT has been established as a standard treatment for high-risk prostate cancer with radiotherapy of up to 70 Gy [3,4]. Bolla et al. reported that in patients with locally advanced disease, the use of goserelin simultaneously with external beam radiotherapy improved the 5-year OS in comparison to external beam RT alone (79% vs. 62%, *p* = 0.001) [5].

ADT is usually used for advanced prostate cancer, including locally advanced and metastatic cancers. However, in Japan, ADT is likely to be accepted even for localized disease [10], because Japanese patients consider mild to moderate toxicity (i.e., sexual dysfunction) acceptable as long as they received curative treatment for lethal cancer, which may reflect the social and philosophical situation in Asian countries [19]. However, the use of primary ADT in localized disease is not a recommended treatment in guidelines [3] because of its possible harmful effect and the lack of benefits for survival [20].

Dose escalation in radiotherapy improved outcomes in several randomized controlled trials and high-BED radiotherapy is recognized as the standard treatment for localized prostate cancer [3,6]. In EBRT, IG-IMRT is now the standard treatment of choice because of its superior dose distribution, which makes it possible to administer high-dose radiotherapy to the lesion without elevating toxicity [3,6]. BT (LDR-BT and HDR-BT) as a ‘‘boost’’ or as monotherapy has also been incorporated as the standard radiotherapy for these excellent dose distributions [21]. These modalities combined with ADT are regarded as the standard treatments for locally advanced and/or intermediate- to high-risk disease; however, there is no high-level evidence that supports higher-dose radiotherapy of ≥74 Gy and/or BT. In addition, the optimum duration of ADT with higher dose RT is yet to be determined [22].

Adverse effects of ADT include decreased bone mineral density; metabolic changes such as weight gain; decreased muscle mass and increased insulin resistance; decreased libido and sexual dysfunction; hot flashes; gynecomastia; reduced testicle size; anemia; and fatigue [4,8]. Several observational studies suggest an increased risk of diabetes and cardiovascular events (myocardial infarction (MI) and sudden cardiac death) [23], although most published studies reported that ADT is not linked to greater cardiovascular mortality, and a meta-analysis of randomized trials in men assigned to ADT vs. no ADT did not record much/excessive discrepancy in cardiovascular mortality between the groups [8]. Studies have also suggested that there may be other harmful effects of ADT, including cerebrovascular diseases [24], kidney injury [25], thromboembolic events [26], and diabetes [23], which may all contribute to excess OCM. ADT significantly increases fat mass and fasting insulin levels and decreases insulin sensitivity [8,23]. Treatment-related changes in serum lipoproteins and arterial stiffness, as well as possible QT interval prolongation, may also contribute to the association between ADT and cardiovascular and cerebrovascular outcomes [23,24].

Unexpectedly, we did not find that ADT had a beneficial effect on survival; furthermore, a negative impact was observed on OCM in the elderly population. In addition, we found several previous retrospective/population-based studies concerning the negative impact of ADT [27,28,29].

Beyer et al. reported that a 10-year OS rate decreased from 44% of hormone naïve case into 20% with ADT [27] in prostate cancer patients who were receiving LDR-BT, with the leading causes of death being cardiovascular, prostate, and other cancers with no obvious discrepancy between the two groups. Abdollah et al. reported that treatment with medical ADT may increase the risk of OCM in 137,524 patients with non-metastatic prostate cancer who were treated between 1995 and 2009; this study was extracted from the Surveillance Epidemiology and End Results Medicare-linked database [28]. Nanda reported that neoadjuvant ADT is significantly associated with an increased risk of all-cause mortality among men with a history of coronary artery disease (CAD)-induced chronic heret falure or MI, but not among men with no comorbidity or a single CAD risk factor, which exceeds death after the LDR-BT of using ADT [29].

Our findings are in line with these findings, and the strength of this study is that we examined the role of ADT on OCM in patients treated with modern high BED radiotherapy focused on age. Morgans et al. speculated that the risk of incident diabetes mellitus (DM) or cardiovascular disease in men exposed to prolonged ADT ≥2 years increases with age at diagnosis and occurred 5–10 years later [30]. They reported that younger men are not at increased risk for incident DM or cardiovascular disease even when treated with ADT ≥2, whereas older men exposed to prolonged ADT are at an increased risk of these illnesses. This finding supports our result that DM and/or cardiovascular disease could increase OCM risk. Recently, the outcomes of prostate cancer treatment has improved and reached nearly a 100% survival rate; therefore, simultaneous importance of OCM has increased. The use of ADT in elderly patients should be performed with meticulous care.

This study has several limitations. First, as it was a retrospective study carried out in a few institutes dealing with a rather small number of patients, a longer follow-up with larger number of patients is needed before making concrete conclusions since there is a chance of an inherent bias due to inhomogeneity remaining. Next, comorbidity analysis is lacking. Body mass index, adrenal dysfunction, metabolic syndrome, and comorbidities, such as DM and cardiovascular disease, are confirmed as important influencing factors for OCM [8,23,24,25]. In addition, lack of serum testosterone measurements is a problem since many of the older men will have a prolonged recovery time after two years of ADT. Lastly, the reason for OCM could not be specified; it is not always the cardiovascular system, i.e., mainly other cancers. This retrospective study may influence the uncorrected bias. At present, no single likely explanation can be provided for the excess deaths. Therefore, our results did not reduce the importance of ADT usage in situations to improve survival in randomized clinical trials.

In conclusion, treatment with ADT is correlated with the risk of mortality due to causes other than prostate cancer, especially in localized prostate cancer patients aged ≥75 years. Whether this is a simple association or a cause-and-effect relationship is unknown and warrants further prospective studies.

## Figures and Tables

**Table 1:** Characteristics and treatment factors of patients according to androgen deprivation therapy (ADT).

Variables	Strata	ADT (+) *n* = 867			ADT (−) *n* = 258	*p* Value
		ADT ≥ 2 Years *n* = 135	ADT < 2 Years *n* = 732	ADT (+) (%)		
		No. or Median (Range)	No. or Median (Range)		No. or Median (Range)	
Age	50	11	67	(68%)	36	**0.0591**
	60	40	279	(75%)	107	
	70	50	222	(80%)	69	
	75	34	164	(81%)	46	
T category	1	11	271	(68%)	133	**<0.0001**
	2	32	367	(77%)	119	
	3	87	90	(97%)	6	
	4	5	4	(100%)	0	
Pretreatment PSA	ng/mL	23.4 (3.19–378)	8.62 (1.4–337)		6.5 (2.34–61)	**<0.0001**
Gleason score	≤6	27	295	(67%)	159	**<0.0001**
	7	35	298	(82%)	75	exc NA
	8≤	58	137	(89%)	23	
	NA	15	2	(94%)	1	
NCCN risk classification	Low	0	145	(54%)	122	**<0.0001**
	Intermediate	19	356	(79%)	100	
	High	116	231	(91%)	36	
IG-IMRT		24	149	(65%)	93	**<0.0001**
BT		111	583	(81%)	165	
	LDR-BT	2	397	(82%)	87	
	HDR-BT	109	186	(79%)	78	
Follow-up	Months	82.9 (14–241)	83.0 (5–192)		77.2 (14.5–161)	**0.0037**

Bold values indicate statistically significance, NA; not available, ADT (+) = ADT ≥2 years + ADT <2 years. OCM = other cause of mortality, BT = LDR-BT+HDR-BT, *p* value depicts comparison between ADT (−) vs. ADT (+).

**Table 2:** Characteristics and treatment factors of patients with overall survival (mortality) according to duration of ADT.

Variables		Strata	Died *n* = 81	Died (+) %	Alive *n* = 1044	*p* Value
			No.		No.	
Total		ADT (−)	10	(4%)	862	**<0.0001**
		ADT < 2Y	48	(7%)	70	
		ADT ≥ 2Y	23	(17%)	112	
NCCN risk classification	Low	ADT (−)	4	(3%)	118	0.7114
		ADT < 2Y	6	(4%)	139	
		ADT ≥ 2Y	0	NA	0	
	Intermediate	ADT (−)	3	(3%)	97	0.3683
		ADT < 2Y	19	(5%)	337	
		ADT ≥ 2Y	2	(11%)	17	
	High	ADT (−)	3	(8%)	33	0.0798
		ADT < 2Y	23	(10%)	208	
		ADT ≥ 2Y	21	(18%)	95	
Age	Young <75	ADT (−)	8	(4%)	204	**0.0017**
		ADT < 2Y	32	(6%)	536	
		ADT ≥ 2Y	14	(14%)	87	
	Elder ≥75	ADT (−)	2	(4%)	44	**0.0050**
		ADT < 2Y	16	(10%)	148	
		ADT ≥ 2Y	9	(26%)	25	

Bold values indicate statistically significance, ADT = androgen deprivation therapy, NA; not available.

**Table 3:** Association between other cause of mortality (OCM) and clinical-pathological characteristics with the patient cohort.

Variables	Strata	OCM (+) *n* = 71	OCM (+) (%)	OCM (−) *n* = 1054	*p* Value
		No. or Median (Range)		No. or Median (Range)	
Age	<60	2	(2%)	112	**0.0017**
	60-69	19	(4%)	407	
	70–74	24	(7%)	317	
	75≤	26	(11%)	218	
T category	1	18	(4%)	397	**0.0004**
	2	29	(6%)	489	
	3	24	(13%)	159	
	4	0	(0%)	9	
iPSA	ng/mL	11.95 (2.68–245)		8.40 (1.4–375)	**0.0151**
Gleason score	≤6	28	(6%)	453	0.9507
	7	25	(6%)	383	exc NA
	8≤	14	(6%)	204	
	NA	4	(22%)	14	
NCCN risk classification	Low	10	(4%)	257	**0.0018**
	Intermediate	23	(5%)	452	
	High	38	(10%)	345	
PSA failure	Yes	1	(1%)	71	0.1272
	No	70	(7%)	983	
ADT	Yes	61	(7%)	797	**0.0422**
	No	10	(4%)	257	
	Duration	6 (0–173)		6 (0–45)	0.0486
IG-IMRT		13	(5%)	253	**<0.0001**
BT		58	(7%)	801	
	HDR-BT	43	(12%)	330	
	*LDR-BT*	*15*	*(3%)*	*471*	

Bold values indicate statistically significance, NA; not available, ADT = androgen deprivation therapy, HDR-BT; high-dose-rate brachytherapy, LDR-BT; low-dose-rate brachytherapy, iPSA = initial PSA, IG-IMRT; image guided intensity modulated radiotherapy, BT = HDR-BT + LDR-BT. exc NA = p value was calculated excluding NA.

**Table 4:** Characteristics and treatment factors of patients with other cause of mortality (OCM) according to duration of ADT.

Variables		Strata	OCM (+) *n* = 71	OCM (+) %	OCM (−) *n* = 1054	*p* Value
			No.		No.	
Total		ADT (−)	10	(4%)	257	**0.0007**
		ADT < 2Y	43	(6%)	680	
		ADT ≥ 2Y	18	(13%)	117	
NCCN risk classification	Low	ADT (−)	4	(3%)	118	0.7126
		ADT < 2Y	6	(4%)	139	
		ADT ≥ 2Y	0	NA	0	
	Intermediate	ADT (−)	2	(2%)	98	0.1945
		ADT < 2Y	19	(5%)	337	
		ADT ≥ 2Y	2	(11%)	17	
	High	ADT (−)	3	(8%)	33	0.2477
		ADT < 2Y	19	(8%)	212	
		ADT ≥ 2Y	16	(14%)	100	
Age	Young <75	ADT (−)	7	(3%)	205	**0.0439**
		ADT < 2Y	28	(5%)	540	
		ADT ≥ 2Y	10	(10%)	91	
	Elder ≥75	ADT (−)	2	(4%)	44	**0.0185**
		ADT < 2Y	16	(10%)	148	
		ADT ≥ 2Y	8	(24%)	26	

Bold values indicate statistically significance, NA; not available, ADT = androgen deprivation therapy, 2Y = 2 years.

**Table 5:** Characteristics and treatment factors of elder patients and younger counterpart.

Variables	Strata	Elder ≥75 *n* = 244		Young <75 *n* = 881		*p* Value
		No. or Median (Range)	(%)	No. or Median (Range)	(%)	
T category	1	73	(30%)	342	(39%)	**0.0219**
	2	127	(52%)	391	(44%)	
	3	44	(18%)	139	(16%)	
	4	0	(0%)	9	(1%)	
Pretreatment PSA	ng/mL	9.74 (1.97–245)		8.32 (1.4–378)		**0.0449**
Gleason score	≤6	82	(34%)	399	(45%)	**0.0004**
	7	93	(38%)	315	(36%)	**exc NA**
	8≤	66	(27%)	152	(17%)	
	NA	3	(1%)	15	(2%)	
NCCN risk classification	Low	36	(15%)	231	(26%)	**0.0005**
	Intermediate	108	(44%)	367	(42%)	
	High	100	(41%)	283	(32%)	
PSA failure	Yes	14	(6%)	58	(7%)	**0.6329**
	No	230	(94%)	823	(93%)	
ADT	Yes	198	(81%)	669	(76%)	**0.0866**
	No	46	(19%)	212	(24%)	
	Duration	6 (0–120)		6 (0–257)		**0.0274**
OCM	Yes	26	(11%)	45	(5%)	**0.0016**
	No	218	(89%)	836	(95%)	
IG-IMRT		83	(34%)	183	(21%)	**<0.0001**
BT		161	(66%)	698	(79%)	
	HDR-BT	91	(37%)	282	(32%)	
	LDR-BT	70	(29%)	416	(47%)	

Bold values indicate statistically significance, NA; not available, ADT = androgen deprivation therapy, HDR-BT; high-dose-rate brachytherapy, LDR-BT; low-dose-rate brachytherapy, iPSA = initial PSA, IG-IMRT; image guided intensity modulated radiotherapy, BT = HDR-BT + LDR-BT, NCCN = The National Comprehensive Cancer Network, OCM = other cause of mortality.

**Table 6:** Cause of other cause of mortality (OCM).

**Total**	**ADT (−)** ***n* = 258**		**ADT < 2Y** ***n* = 732**		**ADT ≥ 2Y** ***n* = 135**	
Cardiovascular			3	(0.4%)	2	(1.5%)
Cerebrovascular			3	(0.4%)	2	(1.5%)
Other malignancies	7	(2.7%)	25	(3.4%)	7	(5.2%)
Other	2	(0.8%)	5	(0.7%)	2	(1.5%)
Unknown	2	(0.8%)	6	(0.8%)	5	(3.7%)
Elder ≥ 75	*n* = 46		*n* = 164		*n* = 34	
Cardiovascular			2	(1.2%)		
Cerebrovascular					1	(2.9%)
Other malignancies	1	(2.2%)	11	(6.7%)	2	(5.9%)
Other	1	(2.2%)	2	(1.2%)	1	(2.9%)
Unknown			1	(0.6%)	4	(11.8%)
Young < 75	*n* = 212		*n* = 568		*n* = 101	
Cardiovascular			1	(0.2%)	2	(2.0%)
Cerebrovascular			3	(0.5%)	1	(1.0%)
Other malignancies	6	(2.8%)	14	(2.5%)	5	(5.0%)
Other	1	(0.5%)	3	(0.5%)	1	(1.0%)
Unknown	2	(0.9%)	5	(0.9%)	1	(1.0%)

ADT = androgen deprivation therapy; 2Y = 2 years.
